# Dopamine and Serotonin Modulate Free Amino Acids Production and Na^+^/K^+^ Pump Activity in Chinese Mitten Crab *Eriocheir sinensis* Under Acute Salinity Stress

**DOI:** 10.3389/fphys.2018.01080

**Published:** 2018-10-10

**Authors:** Zhaoqun Liu, Zhi Zhou, Lingling Wang, Meijia Li, Weilin Wang, Qilin Yi, Shu Huang, Linsheng Song

**Affiliations:** ^1^Liaoning Key Laboratory of Marine Animal Immunology, Dalian Ocean University, Dalian, China; ^2^Functional Laboratory of Marine Fisheries Science and Food Production Processes, Qingdao National Laboratory for Marine Science and Technology, Qingdao, China; ^3^Liaoning Key Laboratory of Marine Animal Immunology and Disease Control, Dalian Ocean University, Dalian, China; ^4^Key Laboratory of Tropical Biological Resources of Ministry of Education, Hainan University, Haikou, China

**Keywords:** *Eriocheir sinensis*, transcriptomic analysis, acute salinity stress, free amino acid, Na^+^/K^+^ ATPase, monoamine neurotransmitters

## Abstract

The Chinese mitten crab *Eriocheir sinensis* lives in saline or fresh water during different life stages and exhibits a complex life history, making it an ideal model to study the salinity adaptation of euryhaline animals. In this study, RNA-seq techniques, and determinations of free amino acids (FAAs), monoamine neurotransmitters, and Na^+^/K^+^ pump activity, were employed to understand the osmoregulatory mechanism in Chinese mitten crab. A total of 15,138 differentially expressed genes were obtained from 12 transcriptome libraries. GO enrichment analysis revealed that the mRNA expression profiles were completely remodeled from 12 to 24 h after salinity stress. The neuroendocrine system was activated under stimulation, and the monoamine neurotransmitters including dopamine (DA) and serotonin (5-HT) were released to modulate osmoregulation. Furthermore, the Na^+^/K^+^ pump in crab hemocytes was significantly inhibited post salinity stress, resulting in increased intracellular ion concentrations and osmotic pressure to sustain the osmotic balance. Moreover, six key FAAs, including alanine (Ala), proline (Pro), glycine (Gly), glutamate (Glu), arginine (Arg), and aspartate (Asp), were overexpressed to modulate the extracellular osmotic balance during salinity adaptation. Interestingly, the immune genes were not enriched in the GO analysis, implying that the immune system might not contribute fundamentally to the tolerance upon fluctuating ambient salinity in the Chinese mitten crab. These results collectively demonstrated that the Chinese mitten crab had evolved an efficient regulation mechanism by modulating the FAAs production and Na^+^/K^+^ pump activity to sustain the osmotic balance independent of the immune system, in which the neuroendocrine modulation, especially generated by the monoamine neurotransmitter, played an indispensable role.

## Introduction

The Chinese mitten crab, *Eriocheir sinensis*, has a unique life cycle. The adults grow in freshwater, while their mating activities take place in brackish water and the larvae hatch in the nearshore area (Li et al., 2014). Therefore, they can survive in waters fluctuating in salinity (Silvestre et al., 2005). More importantly, the superb osmoregulatory capacity of the Chinese mitten crab in some way recapitulates the evolution processes of the ancient creatures, making it an ideal model to study the evolution transition of life from oceans to lands (Li et al., 2014). Unfortunately, the concise osmoregulatory mechanisms in *E. sinensis* are still unclear and deserves further exploration.

Extensive literature has described the complex ways in which the aquatic creatures respond to acute salinity stress at the molecular level (Kultz et al., 2007), among which the free amino acids (FAAs) are proved to be critical for maintaining the cell volume and salinity balance by causing the accumulation of FAAs in the cytoplasma (Yancey et al., 1982; Gilles, 1997; Kempf and Bremer, 1998; McNamara et al., 2004). Among all the FAAs, proline (Pro), alanine (Ala), glutamate (Glu), glycine (Gly), Arginine (Arg), and Aspartate (Asp) are the key contributors in the osmoregulation of crustaceans (Di Martino et al., 2003; Silvia et al., 2004; Watanabe et al., 2005). Apart from the FAAs, Na^+^/K^+^ ATPase is also demonstrated to be responsible for osmoregulation in crabs. It is able to transport Na^+^ out of the cells and K^+^ into the cells on the opposite of their respective concentrations (Skou and Esmann, 1992; Sun et al., 2012). It is the basic modulator of ion transport across the cell membrane in euryhaline crabs (Neufeld et al., 1980). Recently, investigation has been extended to understand the key modulators and pathways regulating the FAAs’ production and specific ion channel activity in crustaceans (Huong et al., 2001).

Accumulating evidences have illustrated that elevated salinity can lead to the release of stress-related hormones and neurotransmitters, thereby modulating energy metabolism and influencing reproduction (Lushchak, 2011). It was reported that monoamines, such as dopamine (DA), serotonin (5-HT), and octopamine, secreted by the neuroendocrine systems of crabs could induce increased Na^+^ influx in a cAMP-dependent fashion (Morris, 2001). Besides, Trausch et al. (1989) found that DA and serotonin (5-HT) could induce protein phosphorylation of Na^+^/K^+^ ATPase in *E. sinensis* (Trausch et al., 1989). By far, most of the previous study mainly focused on Na^+^/K^+^ pump and FAAs production in gill, which is the important tissue responsible for breath and ormoregulation in crabs (Paital and Chainy, 2010). Being the most important immune cells, the circulating hemocytes in mitten crab should also be critical for the homeostasis maintenance during salinity adaptation (Wang et al., 2018). In this paper, transcriptome of *E. sinensis* hemocytes under acute salinity stress was analyzed to reveal the underlying osmoregulatory mechanism. The major purposes are to (1) illustrate the differentially expressed genes from *E. sinensis* hemocytes under acute salinity stress; (2) explore the concentration changes of monoamine neurotransmitters (DA and 5-HT) in crab serum; (3) investigate the potential osmoregulatory mechanisms in crab hemolymph modulated by monoamine neurotransmitters.

## Materials and Methods

### Salinity Stress Experiments and Sample Collection

One hundred and eighty-one-year-old Chinese mitten crabs *E. sinensis*, whose approximate weight was 20 g, were collected from a local farm in Qingdao, Shandong, China. These individuals were cultured in tanks equipped with adequate aeration, temperature (20°C), and food (twice daily) for 2 weeks. All animal-involving experiments of this study were approved by the Ethics Committee of Dalian Ocean University.

The crabs were divided into six groups and each group contained thirty individuals. The crabs in the first group were cultured in fresh water and sampled at 24 h post treatment, and designated as S0_t24 (control) group. In the S16_t12, S16_t24, and S16_t48 groups, the crabs were raised in saline water at a salinity of 16‰ and sampled at 12, 24, and 48 h after incubation. Besides, in the S28_t24 and S35_t24 groups, the crabs were treated with acute salinity stress of 28 and 35‰, respectively, and sampled at 24 h post treatment.

The hemolymph was collected from the crab chelipeds using a syringe with an equal volume of anticoagulant (27 mmol L^-1^ sodium citrate, 336 mmol L^-1^ NaCl, 115 mmol L^-1^ glucose, 9 mmol L^-1^ EDTA, pH 7.0), and centrifuged at 800 *g*, 4°C for 10 min to harvest the hemocytes for the subsequent RNA preparation and ion determination. The serum was frozen immediately with liquid nitrogen for the determination of FAA contents and monoamine neurotransmitter concentrations. Hemocytes from three crabs were sampled as one duplicate, and two duplicates were conducted for the RNA sequencing. Hemocytes and serum from another three crabs were collected as one duplicate for the measurements of FAAs, neurotransmitters, and ion, and three replicates were employed for each assay.

### Total RNA-Seq and Analysis of Differentially Expressed Transcripts

The RNA-seq was conducted according to the description in our previous research (Liu et al., 2017). The data of sequencing are now available on NCBI in the SRA (Short Read Archive) database under the accession number of **SRP131532**. The transcriptome generated through shotgun assembly from the NCBI database (GenBank: GFBL00000000.1) was used as a reference transcriptome for RNA-seq expression analysis. LifeScope^TM^ and “TopHatBowtie” were employed in reads alignment and sequence mapping, while cufflinks was used for transcripts assembling (Trapnell et al., 2012).

### Determination of Monoamine Neurotransmitter Concentrations

The concentrations of monoamine neurotransmitters DA and 5-HT were determined with Elisa kits (Abnova, KA1887, and KA2518). Briefly, the DA and 5-HT were extracted using a *cis*-diol-specific affinity gel, acylated, and then converted enzymatically. The competitive ELISA kit used the microtiter plate format. The antigen was bound to the solid phase of the microtiter plate. The derivatized standards, controls and samples, and the solid phase bound analytes competed for a fixed number of antibody binding sites. Next, washing was performed three times to remove the unbound antibody. Finally, the bound antibody was measured by TMB method.

### Quantification of Intracellular Ion Contents and FAAs in Serum

The intracellular ion (Na^+^, K^+^, and Ca^2+^) contents were assayed in the Testing & Analysis Center, the Institute of Oceanography, Chinese Academy of Sciences, with high-performance liquid chromatography (HPLC) methods. The contents of FAAs in the crab serum were also determined via HPLC in a Cosmosil 5C18AR-2 packed column (Sato et al., 1992).

### Statistical Analysis

Data statistical analyses were performed on SPSS using one-way ANOVA. Values are given as Means ± Standard Error (SE), and a significance level of *p* < 0.05 was used for all tests. ^∗^ was used to represent the significance of *p* < 0.05, while ^∗∗^ was used to represent the significance of *p* < 0.01.

## Results

### The Output of RNA Sequencing

A total of 282,465,912 single end reads were obtained. The read numbers in each library are shown in **Table 1**. After removing the non-coding RNA, the remaining clean reads in each library were mapped to a transcriptome of *E. sinensis* downloaded from NCBI database, and the successfully mapping rates ranged from 59.16 to 85.94% (**Table 1**). The uniquely mapping rates from twelve libraries fluctuated within the range of 2,602,114 to 11,589,761 (**Table 1**).

**Table 1:** The overview of RNA-Seq.

Treatment	Raw	Mapping	Mapping	Unique
	reads	reads	rate	mapping
S0_t24-1	47,764,205	28,257,480	59.16%	11,589,761
S0_t24-2	23,541,188	13,483,571	57.28%	4,158,542
S16_t12-1	26,723,146	21,694,343	81.18%	6,936,988
S16_t12-2	17,347,940	14,613,056	84.23%	5,397,520
S16_t12-3	18,172,364	15,286,169	84.12%	5,381,978
S16_t24-1	20,826,501	16,770,719	80.53%	4,432,099
S16_t24-2	12,680,653	8,537,297	67.34%	3,613,020
S16_t24-3	11,725,476	7,761,731	66.20%	2,602,114
S16_t48-1	22,717,322	18,247,888	80.33%	6,285,361
S16_t48-2	19,967,267	16,802,632	84.15%	5,576,320
S28_t24-1	19,223,318	16,520,546	85.94%	4,871,252
S28_t24-2	13,387,547	11,239,386	83.95%	3,487,126
S35_t24-1	11,601,908	8,912,201	76.82%	2,639,090
S35_t24-2	16,787,077	13,165,014	78.42%	3,544,010


### Identification of the Differentially Expressed Genes

**FIGURE 1:**
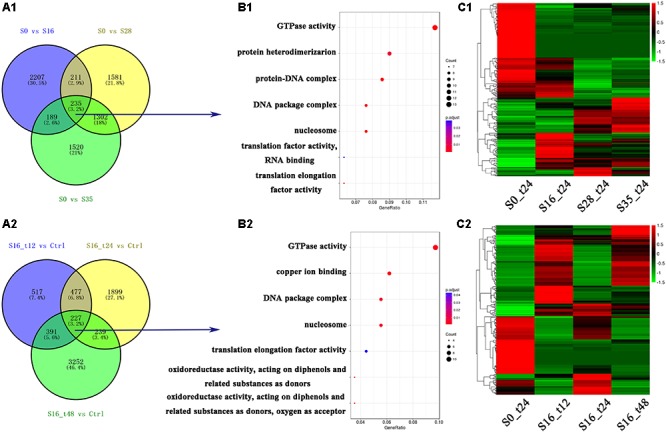
Grouping and enrichment analysis of differentially expressed genes. **(A1)** Grouping of the differentially expressed genes in S16_t24, S28_t24, and S35_t24 groups. **(A2)** Grouping of the differentially expressed genes in S16_t12, S16_t24, and S16_t48 groups. **(B1)** Enrichment analysis of the 235 differentially expressed genes shared by the gene lists in S16_t24, S28_t24, and S35_t24 groups. **(B2)** Enrichment analysis of the 277 differentially expressed genes shared by the gene lists in S16_t12, S16_t24, and S16_t48 groups. **(C1)** Cluster map of the differentially expressed genes in the S16_t24, S28_t24, and S35_t24 groups. **(C2)** Cluster map of the differentially expressed genes in the S16_t12, S16_t24, and S16_t48 groups.

In total, 15,138 differentially expressed genes were obtained, and their numbers were shown in **Figure 1A**. Comparing with the control group (S0_t24), the numbers of differentially expressed genes in the S16_t12, S16_t24, S16_t48, S28_t24, and S35_t24 were 1,612; 2,842; 4,109; 3,329; and 3,246, respectively (**Figures 1A1,A2**). There were 235 differentially expressed genes shared by the S16_t24, S28_t24, and S35_t24 groups, implying their participation in the osmoregulation under different salinities. Meanwhile, there were 277 differentially expressed genes shared by the S16_t12, S16_t24, and S16_t48 groups, reflecting the time course effects of one specific salinity (16‰).

### GO Annotation of Significantly Up-regulated Genes

By analyzing the 235 differentially expressed genes, seven GO terms were identified (**Figure 1B1**), including GTPase (GO: 0003924), protein heterodimerization (GO: 0046982), protein-DNA complex (GO: 0032993), DNA package complex (GO: 0044815), nucleosome (GO: 0000786), translation factor activity, RNA binding (GO: 0003723), and translation elongation factor activity (GO: 0003746). Besides, enrichment analysis of the 277 differentially expressed genes revealed seven GO terms relevant with the time course salinity tolerance mechanism, including GTPase (GO: 0003924), copper ion binding (GO: 0005507), DNA package complex (GO: 0044815), nucleosome (GO: 0000786), elongation factor activity (GO: 0003746), oxidoreductase activity – acting on diphenols and related substances as donors (GO: 0016679), and oxidoreductase activity – acting on diphenols and related substances as donors, oxygen as acceptor (GO: 0016682) (**Figure 1B2**).

The expression profiles of all the differentially expressed genes in the salinity stress groups are shown in **Figure 1C**. The mRNA expression profiles in the S16_t24, S28_t24, and S35_t24 groups were obviously different from that in the S0_t24 group (**Figure 1C1**), while the expression profiles in the S16_t12 and S16_t48 groups were also significantly different from that in the S0_t24 group (**Figure 1C2**).

### Concentration Changes of Monoamine Neurotransmitters in Crab Serum

**FIGURE 2:**
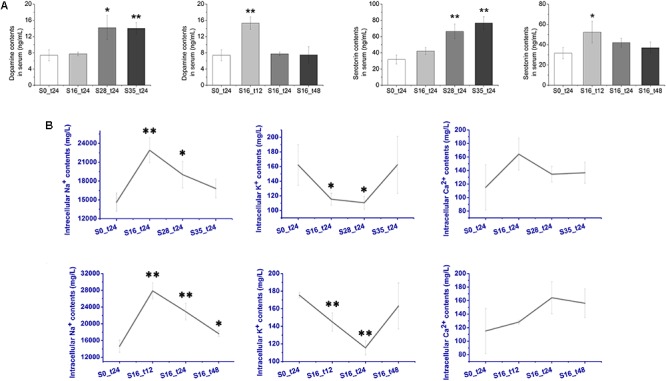
Contents of dopamine (DA), serotonin (5-HT), and intracellular ions after salinity stress. **(A)** Contents of monoamine neurotransmitters DA and 5-HT in crab serum after salinity stress. **(B)** Contents of intracellular Na^+^, K^+^, and Ca^2+^ in crab hemocytes after salinity stress.

The concentration changes of two important monoamine neurotransmitters, DA and 5-HT, were determined after acute salinity stress (**Figure 2A**). The DA contents in the S16_t12 (15.3 ng mL^-1^) and S28_t24 (14.0 ng mL^-1^) groups were extremely higher than that in the S0_t24 group (7.40 ng mL^-1^) (*p* < 0.01). Dopamine concentration in S35_t24 group (14.2 ng mL^-1^) was also dramatically higher (*p* < 0.05). As for 5-HT, its concentrations in the S28_t24 (66.3 ng mL^-1^) and S35_t24 (76.7 ng mL^-1^) groups were extremely higher than that in the S0_t24 group (31.7 ng mL^-1^) (*p* < 0.01). The 5-HT content in the S16_t12 group (52.3 ng mL^-1^) was also significantly up-regulated (*p* < 0.05). No dramatic change of 5-HT concentrations was observed in the S16_t24 and S16_t48 groups (*p* > 0.05).

### The Variation of Intracellular Ion Contents

The intracellular ion (Na^+^, K^+^, and Ca^2+^) contents were assayed using the HPLC method. As shown in **Figure 2B**, the Na^+^ contents increased dramatically from 14,636.67 mg L^-1^ in the S0_t24 group to 22,910.00 mg L^-1^ in the S16_t24 group (*p* < 0.01) after salinity stress, and remained significantly higher in the S28_t24 group (19016.67 mg L^-1^, *p* < 0.05). The Na^+^ contents in the S16_t12 group was 27873.00 mg L^-1^, which was extremely higher (*p* < 0.01), while the Na^+^ contents in the S16_t48 group (17641.00 mg L^-1^) was also significantly higher in comparison with the S0_t24 group (*p* < 0.05). The Na^+^ contents in the S35_t24 group showed no obvious change (*p* > 0.05). As for K^+^, its contents in the S16_t12 and S16_t24 groups were 144.93 and 115.47 mg L^-1^, respectively, which were extremely down-regulated (*p* < 0.01). The K^+^ content in the S28_t24 group (110.60 mg L^-1^) was also obviously lower than that in the control group (*p* < 0.05). No significant change in K^+^ contents was determined in the rest of the groups (*p* > 0.05). Moreover, there was no obvious change in Ca^2+^ contents observed between the control and stress groups (*p* > 0.05).

### Free Amino Acid (FAA) Concentrations and Composition

**FIGURE 3:**
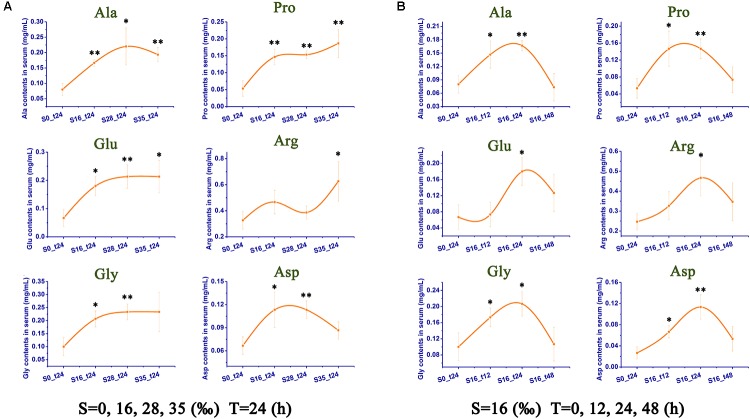
Contents of free amino acids (FAAs) in crab serum after salinity stress. **(A)** Contents of FAAs in serum at 24 h after different salinities stress. **(B)** Contents of FAAs in serum from 12 to 48 h under one specific salinity stress (16‰).

Among all the tested FAAs, six of them were significantly expressed after acute salinity stress, including Ala, Pro, Glu, Arg, Gly, and Asp (**Figure 3**). The contents of these FAAs were increased with the elevation of salinities. As shown in **Figure 3A**, the contents of nearly every FAA was up-regulated to a significantly higher level (*p* < 0.05), some of which rose to an extremely higher extent (*p* < 0.01). Similar trends were also observed in **Figure 3B**, which reflected the time course of osmoregulation at a specific salinity value (16‰). Interestingly, at 48 h after stress, the contents of all the six FAAs restored to the initial level when compared with that in the control group (*p* > 0.05).

### Characterization of the Osmoregulatory Pathway in Crab Hemocytes

**FIGURE 4:**
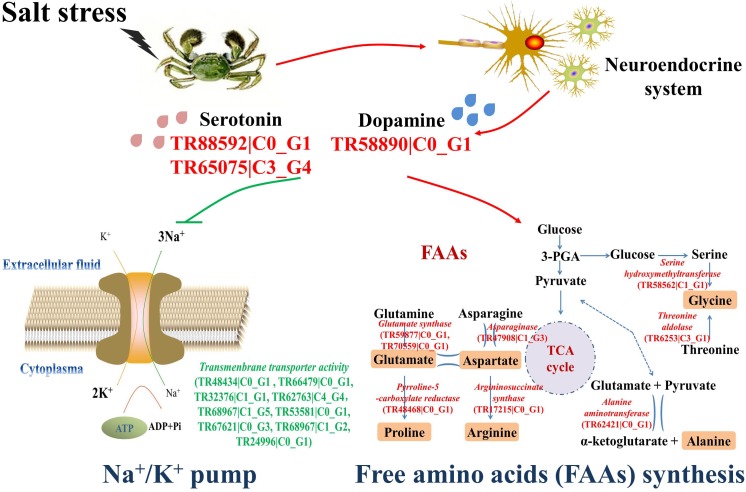
The osmoregulatory pathways in the hemocytes of Chinese mitten crab. The significantly up-regulated genes were shown in green letters, and the significantly up-regulated genes were shown in red letters.

Several differentially expressed genes were characterized from the transcriptomic analysis to illustrate the possible osmoregulatory pathway in crab hemocytes (**Figure 4**). Three genes related to DA and 5-HT synthesis were obviously up-regulated (red), including serotonin receptor (TR88592| C0_G1), serotonin metabolic process–related gene (TR65075| C3_G4), and dopa decarboxylase (TR58890| C0_G1). The mRNA expressions of nine genes (TR48434| C0_G1, TR66479| C0_G1, TR32376| C1_G1, TR62763| C4_G4, TR68967| C1_G5, TR53581| C0_G1, TR67621| C0_G3, TR68967| C1_G2, and TR24996| C0_G1) exhibiting Na^+^/K^+^ transmembrane transporter activity were dramatically inhibited (green). Furthermore, the mRNA expressions of the key synthesizing enzymes of the six salinity-tolerance-related FAAs, such as serine hydroxymethyltransferase (TR58562| C1_G1), threonine aldolase (TR6253| C3_G1), alanine aminotransferase (TR62421| C0_G1), asparaginase (TR47908| C1_G3), glutamate synthase (TR59877| C0_G1, TR70559| C0_G1), pyrroline-5-carboxylate reductase (TR48468| C0_G1), and argininosuccinate synthase (TR17215| C0_G1), were also promoted after stress (red).

## Discussion

The Chinese mitten crab has evolved a successful osmoregulation mechanism to cope with drastic salinity changes ranging from saline water to fresh water, making it a suitable model for the study of salinity adaptation. In the present study, transcriptomic expression of *E. sinensis* hemocytes under acute salinity stress, together with the biological validations such as intracellular ion contents and FAA concentrations in serum, were investigated to reveal the osmoregulatory mechanisms mediated by hemolymph in Chinese mitten crab.

As shown in **Figure 1**, the mRNA expression profiles of the key differentially expressed genes among the three salinity stress groups (S16_t24, S28_t24, and S35_t24) were significantly remodeled, suggesting that different salinities (*S* = 16, 28, and 35‰) could impose a severe influence on the gene expression of the Chinese mitten crabs (**Figures 1A1–C1**). The expression profiles in the S16_t12 and S16_t48 groups were dramatically changed (**Figures 1A2–C2**), which indicated the cumulative effects of salinity on crabs. However, the changes of immune-related genes were rarely identified from the transcriptome, which was consistent with a former study (Li et al., 2014). These results implied that the Chinese mitten crab might have evolved a highly efficient osmoregulatory mechanism, which possibly maintained the physiological homeostasis independent of the immune regulation.

Crabs exercise hormonal control over osmoregulation (Morris, 2001). In the present study, a series of differentially expressed genes of the neuroendocrine system potentially related to the salinity tolerance were characterized from the transcriptome of crab hemocytes. As shown in **Figure 4**, the dopa decarboxylase (TR58890| C0_G1), serotonin receptor (TR88592| C0_G1), and serotonin metabolic process–related genes (TR65075| C3_G4) were significantly overexpressed (red) post challenge, indicating that the production and release of monoamine neurotransmitters DA and 5-HT were prompted. These results were further validated by determining the concentrations of DA and 5-HT in crab serum with Elisa kits (**Figure 2A**). Briefly, both DA and 5-HT contents were dramatically up-regulated at 12 to 24 h post salinity stress. Similar results were also reported in a previous study on crustaceans. The land crab *Gecarcoidea natalis* could release DA and 5-HT to activate NaCl uptake (Morris, 2001). Crustacean hyperglycemic hormone (CHH) and DA were important for the control of the metabolism and osmoregulation in sub-adult shrimp *Litopenaeus vannamei*, and the DA could stimulate hyperglycemia through CHH released from the neuroendocrine XO–SG complex in the eyestalk (Camacho-Jiménez et al., 2017). Thus, results in the current study suggested that the synthesis of monoamine neurotransmitters was activated under acute salinity stress, and these modulators could play critical roles in the osmoregulation in Chinese mitten crab.

Nine genes exhibiting Na^+^/K^+^ transmembrane transporter activity were identified, including TR48434| C0_G1, TR66479| C0_G1, TR32376| C1_G1, TR62763| C4_G4, TR68967| C1_G5, TR53581| C0_G1, TR67621| C0_G3, TR68967| C1_G2, and TR24996| C0_G1 (**Figure 4**). Their mRNA expression levels were all down-regulated after salinity stress, indicating the inhibition of the Na^+^/K^+^ pump. These results were further ascertained by measuring the intracellular ion contents using the HPLC method. The Na^+^ contents in hemocyte cytoplasma were significantly increased in the salinity stress groups, while the intracellular K^+^ contents were obviously decreased in comparison with the control group (**Figure 2B**), thereby demonstrating that the Na^+^/K^+^ pump was inhibited after salinity stress to elevate the Na^+^ concentrations in the cytoplasma of crab hemocytes. The Na^+^/K^+^ pump is extremely important for the sustainability of osmotic balance and electrical activity in the cell, as well as for the transportation of nutrients into the cell (Skou and Esmann, 1992; Sun et al., 2012). A cell’s osmolarity is the sum of the concentrations of the various ion species, proteins, and other organic compounds inside the cell. When the osmolarity outside the cell is higher than that inside the cell, water flows into the cell through osmosis. This can cause the cell to swell up and lyse (Xie and Cai, 2003), during which the Na^+^/K^+^ pump helps to maintain the right concentration of ions. In this research, when the hemocytes have to cope with a hypertonic extracellular status during salinity stress, higher intracellular Na^+^ concentrations will contribute to sustain the osmotic balance and physiological functions of the hemocytes, thus ensuring the host’s inner homeostasis. Therefore, these results illustrated that the Na^+^/K^+^ pump in Chinese mitten crab hemocyte was inhibited to elevate the intracellular Na^+^ concentrations under acute salinity stress, causing a higher osmolarity inside the cell to re-achieve the osmotic balance. It has been found that Ca^2+^, another important intracellular ion, could modulate cell volume change under salinity challenge (McCarty and O’Neil, 1992; Meng et al., 2013). However, no obvious variation of the intracellular Ca^2+^ contents or expression levels of genes related to the Ca^2+^ channel was observed in this study. These results suggested that the Ca^2+^ channel might not be involved in the osmoregulation in crab hemocytes, which was different from that in molluscs. Moreover, it was reported that the Na^+^/K^+^ pump activity could be modulated by monoamine neurotransmitters, such as DA and 5-HT. For example, DA could regulate ionic transport and glycolytic fluxes in the gills of *E. sinensis* (Detaille et al., 1992). In crab *G. natalis*, the Na^+^ uptake and Na^+^/K^+^ ATPase were stimulated by 5-HT via cAMP-mediated phosphorylation (Morris, 2001). All these results indicated that the Na^+^/K^+^ pump rather than the Ca^2+^ channel made an effort to rebuild the osmotic balance under hyper-osmotic stress, and the inhibition of the Na^+^/K^+^ channel might be modulated by DA and 5-HT released from the neuroendocrine system in *E. sinensis*. In addition, the concentration variations of serotonin and dopamine were not the same, indicating that their functions in the osmoregulation of crab might be different. According to a previous study, dopamine was proved to increase the level of cAMP in the gill of crab, which then increased the sodium uptake and Na^+^/K^+^ ATPase activity (Sommer and Mantel, 1991). In this research, the contents of dopamine were significantly upregulated under salinity stress, and the Na^+^/K^+^ pump was also activated. These results suggested that the Chinese mitten crab might release DA in response to salinity stress and increase the Na^+^/K^+^ ATPase activity by upregulating the intracellular cAMP concentration. However, the regulation mechanism of 5-HT on osmoregulation in crabs have not been reported so far, and this will be further explored in our future study.

Moreover, the mRNA expressions of the key synthesizing enzymes for the six FAAs [alanine (Ala), proline (Pro), glutamate (Glu), arginine (Arg), glycine (Gly), and aspartate (Asp)], including serine hydroxymethyltransferase (TR58562|C1_G1), threonine aldolase (TR6253|C3_G1), alanine aminotransferase (TR62421|C0_G1), asparaginase (TR47908|C1_G3), glutamate synthase (TR59877|C0_G1, TR70559|C0_G1), pyrroline-5-carboxylate reductase (TR48468|C0_G1), and argininosuccinate synthase (TR17215|C0_G1), were also found to be significantly up-regulated (**Figure 4**). Their contents in the crab serum under challenge were also dramatically increased as determined by HPLC (**Figure 3**). According to previous research, D-Alanine was a critical osmoregulator in invertebrates such as crabs and oysters (Matsushima et al., 1984; Okuma and Abe, 1994; Fujimori and Abe, 2002). Organic osmolytes, such as proline were proved to modulate the cell volume in nearly all kinds of creatures (Willett and Burton, 2002). Arginine, together with the other non-essential AAs such as glycine and taurine, work as predominant effectors of intracellular isosmotic adjustment in euryhaline marine decapods (McNamara et al., 2004). These results collectively suggested that the FAAs, such as Ala, Pro, Glu, Gly, Arg, and Asp were important modulators for the osmoregulation in hemocytes of Chinese mitten crab. Until now, no exploration has ever been conducted to understand whether this process was modulated by monoamine neurotransmitters similar to Na^+^/K^+^ pump, which should be a promising area in the future study.

Taken together, the osmoregulatory mechanism in hemocytes of the Chinese mitten crab *E. sinensis* was investigated using the RNA-seq in this article. The Na^+^/K^+^ pump activity was inhibited under salinity stress to elevate the intracellular ion concentrations and osmotic pressure, while the FAA contents in serum were also increased to sustain the extracellular osmotic balance. Moreover, the contents of DA and 5-HT in crab serum were significantly up-regulated after stress, implying that the osmoregulation in Chinese mitten crab might be regulated by the monoamine neurotransmitters.

## Author Contributions

LS, LW, ZL, ZZ, QY, and SH conceived and designed the experiments. ZL carried out the experiments. ZL and ZZ analyzed the data. LS and LW contributed reagents, materials, and analysis tools. ZL, LW, ML, WW, and LS wrote the manuscript. All the authors read and approved the final manuscript.

## Conflict of Interest Statement

The authors declare that the research was conducted in the absence of any commercial or financial relationships that could be construed as a potential conflict of interest.
